# Educational inequalities in hypothermia mortality in the Baltic countries and Finland in 2000–15

**DOI:** 10.1093/eurpub/ckad062

**Published:** 2023-04-24

**Authors:** Andrew Stickley, Aleksei Baburin, Domantas Jasilionis, Juris Krumins, Pekka Martikainen, Naoki Kondo, Jae Il Shin, Hans Oh, Kyle Waldman, Mall Leinsalu

**Affiliations:** Stockholm Centre for Health and Social Change, Södertörn University, Huddinge, Sweden; Department of Social Epidemiology, Graduate School of Medicine and School of Public Health, Kyoto University, Kyoto, Japan; Department of Epidemiology and Biostatistics, National Institute for Health Development, Tallinn, Estonia; Max Planck Institute for Demographic Research, Rostock, Germany; Demographic Research Centre, Vytautas Magnus University, Kaunas, Lithuania; Demography Unit, Faculty of Business, Management and Economics, University of Latvia, Riga, Latvia; Max Planck Institute for Demographic Research, Rostock, Germany; Population Research Unit, University of Helsinki, Helsinki, Finland; Department of Social Epidemiology, Graduate School of Medicine and School of Public Health, Kyoto University, Kyoto, Japan; Department of Pediatrics, Yonsei University College of Medicine, Seoul, Korea; Suzanne Dworak-Peck School of Social Work, University of Southern California, Los Angeles, CA, USA; Department of Sociology, Harvard University, Cambridge, MA, USA; Stockholm Centre for Health and Social Change, Södertörn University, Huddinge, Sweden; Department of Epidemiology and Biostatistics, National Institute for Health Development, Tallinn, Estonia

## Abstract

**Background:**

Despite an increased focus on cold-related mortality in recent years, there has been comparatively little research specifically on hypothermia mortality and its associated factors.

**Methods:**

Educational inequalities in hypothermia mortality among individuals aged 30–74 in the Baltic countries (Estonia, Latvia, Lithuania) and Finland in 2000–15 were examined using data from longitudinal mortality follow-up studies of population censuses (the Baltics) and from a longitudinal register-based population data file (Finland).

**Results:**

Age-standardized mortality rates (ASMRs) were much higher in the Baltic countries than in Finland across the study period. From 2000–07 to 2008–15, overall ASMRs declined in all countries except among Finnish women. Although a strong educational gradient was observed in hypothermia mortality in all countries in 2000–07, inequalities were larger in the Baltic countries. Between 2000–07 and 2008–15, ASMRs declined in all educational groups except for high-educated women in Finland and low-educated women in Lithuania; the changes however were not always statistically significant. The absolute mortality decline was often larger among the low educated resulting in narrowing absolute inequalities (excepting Lithuania), whereas a larger relative decline among the high educated (excepting Finnish women) resulted in a considerable widening of relative inequalities in hypothermia mortality by 2008–15.

**Conclusion:**

Although some reduction was observed in absolute educational inequalities in hypothermia mortality in 2000–15, substantial and widening relative inequalities highlight the need for further action in combatting factors behind deaths from excessive cold in socioeconomically disadvantaged groups, including risky alcohol consumption and homelessness.

## Introduction

Cold temperature is an important source of mortality. In 2000–19, there were an estimated 4.6 million cold-related deaths annually worldwide,^1^ with low temperature being linked to a variety of different causes of death including cardiovascular and respiratory diseases, nervous disorders and external causes.^2^^,^^3^ Despite increasing recognition that cold exposure represents a considerable public health burden,^4^ until now, much of the research has focused on the overall effects of cold temperature, where cold can also be a contributory/indirect cause of death (as in cardiovascular and respiratory deaths) and there has been comparatively little focus on deaths directly attributable to cold (i.e. from accidental hypothermia), where the body’s core temperature falls to <95°F [<35°C].^5^ This may be an important omission. Although these deaths constitute only a small percentage of all cold-related excess deaths,^6^ a recent report found that hypothermia death rates had risen in the United States in the period from 2003 to 2013.^7^ Importantly, hypothermia deaths are preventable deaths^8^ that may disproportionately affect vulnerable subpopulations (e.g. people affected by homelessness, social isolation and/or mental illness),^7^^,^^9^ and thus constitute a potentially modifiable source of health inequality.

This study will examine the role of educational differences in mortality from hypothermia (exposure to excessive cold) in the Baltic countries (Estonia, Latvia and Lithuania) and Finland. To the best of our knowledge, until now, there has been an absence of studies on the potential role of education in hypothermia mortality. However, research on the association between temperature and all-cause mortality has produced conflicting findings for education (showing both educational differences and no association between educational attainment and cold-related deaths).^10^^,^^11^ Studies that have examined the role of possible socioeconomic status (SES) differences in hypothermia deaths using non-education markers of SES have also produced conflicting findings. Specifically, research undertaken in New York City in 2009–19 showed no association between zip code poverty rates and hypothermia-related mortality,^12^ whereas another study undertaken in the same location in the cold season (October–April) in 2005–14 reported more hypothermia deaths in higher poverty neighbourhoods.^9^ In addition, a study that analyzed data for the USA in 2006–10 found that age-adjusted hypothermia mortality rates for counties in the lowest median household income quartile were around two times higher than those for counties in the highest income quartile.^13^ Overall, variability in the results on social determinants of hypothermia deaths may relate to measurement choices (e.g. individual vs. area-based measures of social disadvantage), or differences in the study context (e.g. the level of segregation, quality of housing stock or geographical location).

The Baltic countries and Finland provide an interesting point of comparison. After regaining independence in 1991, the Baltic countries became members of the European Union (EU) in 2004, although their per capita gross domestic product (GDP) remained 2.5–3 times lower than in Finland even at the end of our study period in 2015.^14^ Importantly, the transition years have been marked by a variety of social problems in the Baltic countries—such as increased poverty and a growth in homelessness^15^^,^^16^ that might be relevant for hypothermia mortality. Moreover, although annual mean temperatures are higher in the Baltic countries than in Finland [e.g. 44.1–46.1°F (6.7–7.8°C) vs. 37.3°F (2.9°C) in 2007], all of these countries are cold in the winter months (November to March) as indicated by the fact that average temperatures were below freezing in Finland in all of these months in the 1991–2020 period and in three and four of these months in Latvia/Lithuania and Estonia, respectively.^17^ Indeed, cold temperature has recently been linked to an increased risk of all-cause mortality in both Estonia (Tallinn in 1997–2015) and Latvia (Riga in 2009–15) in the winter months,^18^ while earlier forensic-medical data from Lithuania indicated that the death rate from hypothermia may be comparatively high.^19^

The aim of this study was to examine changes and educational inequalities in hypothermia mortality in the Baltic countries and in Finland in 2000–15. We first assessed changes in overall hypothermia mortality. Then we evaluated changes in hypothermia mortality in different educational groups and how these changes affected inequalities in hypothermia mortality across the study period. A unique aspect of the study is the highly comparable register-based data with reliable individual-level information on attained educational level.

## Methods

### Data

Data for the Baltic countries were obtained from longitudinal mortality follow-up studies of population censuses in 2000 (2001 in Lithuania) and 2011 involving all permanent residents. The censuses combined traditional survey-based enumeration (the share of coverage ranged from 91% in Latvia to 98% in Estonia) and register-based enumeration.^20^ The register-based data did not include information about SES and were therefore excluded from the analysis. All individuals were followed from the census date until the date of death or emigration, or until the end of the follow-up period. The date and cause of death were linked from national mortality registries by National Statistical Offices. Corresponding data for Finland were obtained from the longitudinal register-based population data file of Statistics Finland covering all permanent residents. Data were originally organized into four sub-periods: 2000–03, 2004–07, 2008–11 and 2012–15. The population exposures for those aged 30 years and older were estimated by adding up the number of person years lived by individuals within each 5-year interval age group in a given period. Deaths were allocated to age intervals using the age at death. Data were anonymized and aggregated into multidimensional frequency tables combining deaths and population exposures split by study periods and socio-demographic variables before they were delivered for research purposes.

### Measures

Following the lead of a previous study^7^ deaths from hypothermia were classified using the code X31 (exposure to excessive natural cold) from the 10th revision of the International Classification of Diseases (ICD-10). Socio-demographic data are census-based and were coded by Statistical Offices following a common study protocol. Educational level was categorized using the International Standard Classification of Education (ISCED) 2011.^21^ ‘Low’ education refers to primary and lower secondary education (ISCED categories 0–2), ‘middle’ education covers upper secondary and post-secondary non-tertiary education (categories 3–4) and ‘high’ education includes tertiary education (categories 5–8). The percentage of missing values for education was low (0–0.7%) and these cases were excluded from the analysis (table 1). To ensure that a complete educational history was covered this study focused on the population aged 30–74 years.

**Table 1:** Characteristics of the study populations and ASMRs per 100 000 person years for hypothermia in 2000–15 in the 30–74 age group

Sex	Country	Period	Deaths (*N*)	Person years (*N*)	Educational level				Mortality from hypothermia		*P*-value
					High (%)	Middle (%)	Low (%)	Missing (%)			
									ASMR (95% CI)	Change (%)	
Men	Finland	2000–07	387	11 597 283	26.8	39.3	33.9	0.0	3.2 (2.9–3.6)		
		2008–15	275	11 963 418	28.7	44.5	26.9	0.0	2.1 (1.9–2.4)	−1.1 (−34.8)	<0.000
	Estonia	2000–07	575	2 540 646	25.4	48.7	25.2	0.7	23.0 (21.1–24.9)		
		2008–15	285	2 634 549	27.6	51.2	20.7	0.5	10.7 (9.5–12.0)	−12.3 (−53.4)	<0.000
	Latvia	2000–07	797	3 989 498	15.3	58.2	25.9	0.6	20.1 (18.8–21.6)		
		2008–15	561	3 927 536	18.3	62.4	19.1	0.3	14.2 (13.0–15.4)	−6.0 (−29.7)	<0.000
	Lithuania	2001–07	1098	5 836 369	16.3	59.8	23.3	0.5	19.7 (18.5–20.9)		
		2008–15	1144	6 463 171	19.3	61.0	19.4	0.3	17.7 (16.7–18.7)	−2.0 (−10.0)	0.013
Women	Finland	2000–07	76	11 834 081	31.3	36.2	32.5	0.0	0.6 (0.5–0.8)		
		2008–15	82	12 111 012	36.5	40.1	23.4	0.0	0.6 (0.4–0.7)	−0.0 (−6.7)	0.667
	Estonia	2000–07	161	3 138 593	34.7	44.9	19.9	0.5	5.0 (4.3–5.9)		
		2008–15	56	3 132 532	40.0	46.2	13.4	0.3	1.6 (1.2–2.1)	−3.4 (−68.0)	<0.000
	Latvia	2000–07	303	5 073 222	19.0	58.7	21.9	0.4	5.7 (5.1–6.4)		
		2008–15	170	4 854 352	26.1	60.0	13.7	0.2	3.2 (2.8–3.8)	−2.5 (−43.4)	<0.000
	Lithuania	2001–07	440	7 018 533	19.0	59.3	21.2	0.5	6.2 (5.6–6.8)		
		2008–15	421	7 677 380	24.7	60.4	14.6	0.3	5.1 (4.6–5.6)	−1.1 (−18.2)	0.004

Notes: The follow-up in the first period started from the census date in the Baltic countries, i.e. 31 March 2000 in Estonia, 1 March 2000 in Latvia and 6 April 2001 in Lithuania; in 2008 the follow-up started on 1 January: the follow-up ended on 31 December in respective periods. Change and *P-*values of the differences are calculated in comparison with the first period.

ASMR, age-standardized mortality rate per 100 000 person years; CI, confidence interval.

### Statistical analysis

For the analyses, the data were combined into two sub-periods to increase the statistical power: 2000–07 and 2008–15. The two sub-periods represent distinct economic phases—the first was marked by rapid economic growth, whereas the second began with a brief, but deep recession (the ‘Great Recession’) that was followed by a period of economic stabilization. Age-standardized mortality rates (ASMRs) per 100 000 person years, stratified by sex, were calculated using the European Standard Population.^22^ Absolute educational inequalities in hypothermia mortality were assessed by ASMR differences between low- and high-educated individuals. Relative inequalities were assessed by age-adjusted mortality rate ratios (RR) calculated using Poisson regression. ASMRs and RRs are presented together with 95% confidence intervals (CI). Statistical testing of ASMR differences between study periods was performed using two-tailed tests and exact *P-*values were added to the tables; the level of statistical significance was set at *P *<* *0.05. To assess the magnitude and direction of the potential bias related to the exclusion of register-based data from census records in the Baltic countries, we performed a sensitivity analysis for Latvia comparing ASMRs for hypothermia mortality while including and excluding register-based data. Statistical analyses were performed using STATA 14.2 (Stata Corp., College Station, TX, USA).

## Results

In total, the study included 6831 deaths from hypothermia and covered about 104 million person years of follow-up. The percentage of high educated increased in all countries across the study period (table 1). Among men, ASMRs were more than six times higher in the Baltic countries than in Finland in 2000–07 ranging from 19.7 (Lithuania) to 23.0 (Estonia) per 100 000 person years. Among women, ASMRs were more than eight times higher in the Baltic countries than in Finland ranging from 5.0 (Estonia) to 6.2 (Lithuania) per 100 000. Between 2000–07 and 2008–15, ASMRs declined in all countries except among Finnish women. In the Baltic countries, both the absolute and relative reduction was largest in Estonia and smallest in Lithuania.

Among men, a clear educational gradient was observed in hypothermia mortality in all studied countries in 2000–07 (table 2). Among the high educated, ASMRs ranged from 1.5 (Finland) to 8.4 (Estonia) per 100 000; for the mid educated from 3.3 (Finland) to 23.3 (Estonia) and for the low educated from 4.4 (Finland) to 36.5 (Estonia and Latvia) per 100 000. When compared with the high educated, the mortality RR of the mid educated ranged from 2.25 (Lithuania) to 2.79 (Estonia); the corresponding RR for the low educated ranged from 3.01 (Finland) to 5.61 (Latvia). From 2000–07 to 2008–15, ASMRs decreased significantly in all educational groups in all countries except among the mid and low educated in Lithuania. In absolute terms, the ASMRs declined more among the low educated (except in Lithuania), whereas the relative decline was largest among the high educated. As a result, absolute educational inequalities in hypothermia mortality narrowed (except in Lithuania) but relative inequalities increased substantially in all countries. In 2008–15, the mortality RR for the mid educated ranged from 2.95 (Finland) to 4.52 (Latvia) and for the low educated from 3.87 (Finland) to 9.06 (Latvia).

**Table 2:** ASMRs per 100 000 person years by educational level and mortality RRs for hypothermia in 2000–15 among men in the 30–74 age group

Country	Educational level	ASMR (95% CI)		Change (%)	*P*-value	RR (95% CI)	
		2000–07	2008–15			2000–07	2008–15
Finland	High	1.5 (1.1–2.0)	0.8 (0.5–1.1)	−0.7 (−45.5)	0.013	1	1
	Mid	3.3 (2.8–4.0)	2.3 (1.9–2.8)	−1.0 (−30.0)	0.006	2.38 (1.70–3.35)	2.95 (1.98–4.40)
	Low	4.4 (3.8–5.1)	3.1 (2.5–3.7)	−1.3 (−30.5)	0.004	3.01 (2.16–4.19)	3.87 (2.59–5.79)
	*Diff.*	*2.9*	*2.3*				
Estonia	High	8.4 (6.3–11.0)	3.3 (2.1–4.8)	−5.2 (−61.2)	0.000	1	1
	Mid	23.3 (20.4–26.5)	10.0 (8.4–12.0)	−13.3 (−56.9)	0.000	2.79 (2.09–3.74)	3.04 (1.99–4.64)
	Low	36.5 (31.7–41.8)	24.6 (20.1–29.8)	−11.9 (−32.5)	0.001	4.35 (3.24–5.83)	7.09 (4.64–10.84)
	*Diff.*	*28.1*	*21.3*				
Latvia	High	6.1 (4.4–8.4)	3.1 (1.9–4.6)	−3.0 (−49.6)	0.011	1	1
	Mid	17.0 (15.3–18.9)	13.8 (12.4–15.4)	−3.2 (−18.5)	0.008	2.71 (1.96–3.76)	4.52 (2.96–6.90)
	Low	36.5 (32.5–40.9)	28.8 (24.4–33.7)	−7.7 (−21.2)	0.014	5.61 (4.04–7.78)	9.06 (5.87–13.98)
	*Diff.*	*30.4*	*25.7*				
Lithuania	High	7.8 (6.1–9.9)	5.5 (4.3–6.9)	−2.4 (−30.0)	0.040	1	1
	Mid	17.4 (15.8–19.0)	17.1 (15.8–18.5)	−0.3 (−1.7)	0.779	2.25 (1.76–2.87)	3.12 (2.44–4.00)
	Low	34.6 (31.0–38.6)	33.7 (29.9–37.8)	−1.0 (−2.7)	0.734	4.35 (3.38–5.59)	5.95 (4.59–7.71)
	*Diff.*	*26.8*	*28.2*				

Notes: Change and *P-*values of the differences are calculated in comparison with the first period.

ASMR, age-standardized mortality rate per 100 000 person years; RR, rate ratio; CI, confidence interval; diff., difference between the low and high educated.

Among women, similarly to men, there was a distinct educational gradient in hypothermia mortality in 2000–07 (table 3). Among the high educated, ASMRs ranged from 0.2 (Finland) to 2.1 (Estonia) per 100 000; corresponding ASMRs for the mid educated ranged from 0.6 (Finland) to 5.6 (Lithuania) and for the low educated from 1.0 (Finland) to 16.0 (Latvia). When compared with the high educated, mortality RRs ranged from 2.45 (Estonia) to 3.60 (Finland) for the mid educated and from 4.59 (Estonia) to 8.52 (Latvia) for the low educated. Although from 2000–07 to 2008–15, ASMRs decreased in all educational groups in all countries except among high-educated women in Finland and among low-educated women in Lithuania, statistically significant changes were observed only among high- and mid-educated women in Estonia, and among mid- and low-educated women in Latvia. In Estonia and Latvia, the ASMR decline was larger among the lower educated leading to narrowing absolute inequalities whereas absolute inequalities increased in Lithuania. The relative decline in all Baltic countries was largest among the high educated resulting in a substantial widening of relative inequalities in hypothermia mortality. In 2008–15, mortality RRs for the mid educated ranged from 2.88 (Estonia) to 3.80 (Latvia) and for the low educated from 8.08 (Estonia) to 10.30 (Latvia). In Finland, both absolute and relative inequalities in hypothermia mortality declined.

**Table 3:** ASMRs per 100 000 person years by educational level and mortality RRs for hypothermia in 2000–15 among women in the 30–74 age group

Country	Educational level	ASMR (95% CI)		Change (%)	*P*-value	RR (95% CI)	
		2000–07	2008–15			2000–07	2008–15
Finland	High	0.2 (0.1–0.4)	0.4 (0.3–0.7)	0.3 (168.8)	0.035	1	1
	Mid	0.6 (0.4–0.9)	0.4 (0.2–0.6)	−0.2 (−37.1)	0.147	3.60 (1.48–8.76)	0.93 (0.50–1.73)
	Low	1.0 (0.7–1.4)	0.9 (0.6–1.4)	−0.0 (−5.1)	0.857	5.88 (2.44–14.18)	2.10 (1.19–3.72)
	*Diff.*	*0.8*	*0.5*				
Estonia	High	2.1 (1.3–3.1)	0.6 (0.3–1.2)	−1.5 (−71.9)	0.002	1	1
	Mid	5.3 (4.2–6.7)	1.6 (1.0–2.4)	−3.7 (−70.0)	0.000	2.45 (1.54–3.88)	2.88 (1.30–6.36)
	Low	10.1 (7.0–14.1)	6.9 (3.9–11.3)	−3.1 (−31.0)	0.219	4.59 (2.81–7.50)	8.08 (3.51–18.55)
	*Diff.*	*8.0*	*6.3*				
Latvia	High	1.5 (0.9–2.5)	0.8 (0.4–1.5)	−0.7 (−45.5)	0.152	1	1
	Mid	4.7 (4.0–5.6)	3.2 (2.6–3.9)	−1.5 (−32.5)	0.003	3.03 (1.78–5.15)	3.80 (2.04–7.09)
	Low	16.0 (12.6–19.9)	10.5 (7.4–14.5)	−5.4 (−34.0)	0.033	8.52 (4.95–14.64)	10.30 (5.34–19.86)
	*Diff.*	*14.5*	*9.7*				
Lithuania	High	1.9 (1.2–2.8)	1.5 (0.9–2.2)	−0.4 (−20.9)	0.423	1	1
	Mid	5.6 (4.9–6.4)	4.8 (4.2–5.5)	−0.8 (−14.2)	0.107	2.92 (1.93–4.42)	3.28 (2.21–4.84)
	Low	15.0 (11.9–18.7)	16.3 (12.9–20.3)	1.3 (8.5)	0.610	6.54 (4.25–10.08)	9.48 (6.25–14.38)
	*Diff.*	*13.1*	*14.8*				

Notes: Change and *P-*values of the differences are calculated in comparison with the first period.

ASMR, age-standardized mortality rate per 100 000 person years; RR, rate ratio; CI, confidence interval; Diff., difference between the low and high educated.

Results from the sensitivity analyses showed that by excluding register-only-based data, we have somewhat underestimated hypothermia mortality in Latvia, although the effect on mortality change between periods was not statistically significant (see online Supplementary table S1).

## Discussion

This study used longitudinal register-based follow-up data to examine changes and educational inequalities in hypothermia mortality in the Baltic countries and Finland in 2000–15. Across the study period, ASMRs were much higher in the Baltic countries than in Finland. From 2000–07 to 2008–15, overall ASMRs declined in all countries except among Finnish women. Although a strong educational gradient in hypothermia mortality was found in all countries in 2000–07, inequalities were substantially larger in the Baltic countries. Between 2000–07 and 2008–15, ASMRs declined in all educational groups except for among high-educated women in Finland and low-educated women in Lithuania; the changes in ASMRs were not always statistically significant. The absolute mortality decline was often larger among the low educated resulting in narrowing absolute educational inequalities (except in Lithuania), whereas a larger relative decline among the high educated (except among Finnish women) resulted in a considerable widening of relative inequalities in hypothermia mortality by 2008–15.

Hypothermia ASMRs were significantly higher in the Baltic countries than in Finland across the study period. It is possible that various factors might account for this difference. For example, previous research has indicated that homelessness is an important risk factor for cold-related mortality.^7^^,^^9^ This may be relevant as homelessness is very low in Finland by international standards and there has been a reduction in long-term homelessness in recent years.^23^ However, the situation is very different in the Baltic countries where homelessness has grown in the post-Soviet period fuelled among other things by unemployment and increasing poverty.^15^^,^^16^ Moreover, a recent report from Lithuania has also highlighted that the stringent criteria for entering shelters for the homeless (e.g. having a medical certificate confirming an absence of tuberculosis, not being intoxicated) may have resulted in hypothermia deaths in the winter months.^16^ In addition, hypothermia deaths can also occur indoors^9^^,^^24^ as a result of inadequate/no home heating,^9^^,^^13^ which might also be a factor in the difference. Specifically, there is some evidence that a much larger proportion of individuals in Latvia and Lithuania may have problems adequately heating their homes compared with those in Finland^25^^,^^26^, while an earlier study suggested that a lack of home heating might play a role in some hypothermia deaths in the Baltic countries during very cold winters.^27^

There was a clear educational gradient in excessive cold mortality across the study period with lower educated groups being at significantly increased risk of death. Education might be important for hypothermia mortality in several different ways. For example, low education has been linked with the risk of homelessness in Lithuania,^16^ while a recent study from Australia showed that lower education is associated with an increased risk of being unable to heat one’s home, and that this risk may persist across time.^28^ Education might also be important for the cold-mortality relationship through its association with health lifestyles/behaviours.^29^ In particular, there is some evidence that lower education is linked to a higher likelihood of engaging in risky/harmful alcohol consumption in the Baltic countries and Finland.^30–32^ This may be significant as alcohol can have a detrimental effect on both physiological functioning (prompting rapid cooling) and cognitive ability (impairing decision making),^24^ while various studies have shown that alcohol use and intoxication/alcoholism often play a role in deaths from excessive cold.^9^^,^^13^^,^^33^ Indeed, forensic data from Estonia at the end of the study period in 2014 and 2015 seemingly confirms the important role of alcohol in this context as between 45.9 and 51.1% of individuals who died of hypothermia were intoxicated to some degree (from low to high intoxication).^34^ Finally, lower education has been associated with poorer mental health in the Baltic countries,^35^ while psychiatric ill health is also a risk factor for cold mortality.^7^^,^^9^

Although hypothermia mortality declined in nearly all of the country groups in 2008–15, the relative decline was almost always larger in the highest educated group leading to a widening in hypothermia mortality RRs by the end of the study period. In contrast, the absolute decline was often larger among the low educated leading to a narrowing absolute mortality gap. It is unclear what underlies the reduction observed in the overall number of hypothermia deaths although it can be speculated that a combination of factors might be involved. An earlier study of extreme climatic events in Latvia showed that despite comparatively cold winters in 2009/10 and 2010/11 there had been a decrease in the number of extreme cold days in the period from 1950 to 2010,^36^ while there is also some evidence that temperatures may have continued to rise in these countries in more recent years.^17^ It is possible that the effects of temperature change on cold-related deaths may have also been enhanced by improved medical treatment of hypothermia resulting from the greater availability and safety of rewarming methods observed elsewhere.^5^ Specific anti-cold measures that have been implemented in some of these countries such as providing financial aid for energy in Lithuania may have also had an impact.^37^ Despite the overall reduction in hypothermia mortality, more research is now needed to determine why the extent of the decline has varied between different educational groups. Specifically, whether this might be linked to factors such as education-related disparities in the degree of cold exposure in occupational and leisure-time activities,^38^ result from differences in access to information that has been provided about how to avoid cold risks^37^ between different educational groups, or be related to selective improvements in housing stock. Although decreased absolute educational inequalities indicate some progress in reducing hypothermia mortality among the low educated, substantial and increasing relative inequalities warrant more in-depth studies looking at the context of broader societal factors such as poverty and inequality and their impact on living conditions, health and health behaviours.

The major strength of this study is its use of harmonized longitudinal register-based mortality follow-up data from four countries to examine educational inequalities in hypothermia mortality over a 15-year period. To the best of our knowledge, this is the first study to examine educational inequalities in hypothermia deaths. However, the study also has some limitations. Our sensitivity analysis showed that by excluding register-only-based data from the analyses, we have underestimated the level of hypothermia mortality in Latvia, although the impact on mortality change was not significant. This is an indication that hypothermia deaths may be more common among marginal groups (e.g. the homeless) who are less likely to be enumerated at census. As the likelihood of becoming homeless can be larger among the low educated, we may have also underestimated educational inequalities in hypothermia mortality. In addition, to ensure a more accurate estimation of educational level, the upper age limit of the study was 74 years. This may have resulted in some hypothermia deaths being excluded as research from the USA has indicated that hypothermia mortality rates rise sharply after age 75 years.^13^ Finally, other measures of SES besides education, such as income and financial hardship/poverty that are often associated with educational attainment might have also been important for hypothermia mortality in these countries. However, we were not able to obtain information on these phenomena to examine their potential impact.

In conclusion, this study showed that hypothermia mortality rates were much higher in the Baltic countries compared with Finland. Lower educated men and women had much higher mortality compared with their higher educated counterparts in all the studied countries. Hypothermia mortality declined in all countries and in nearly all educational groups across the 2000–15 period. Although some reduction was observed in absolute educational inequalities across the study period, widening relative inequalities in hypothermia mortality highlight the need for further action in combatting factors behind deaths from excessive cold in socioeconomically disadvantaged groups, including risky alcohol consumption and homelessness.

## Supplementary Material

ckad062_Supplementary_Data

## Data Availability

The data that support the findings of this study are available from National Statistical Offices, i.e. Statistics Estonia, Statistics Lithuania, Central Statistical Bureau of Latvia and Statistics Finland but restrictions apply on the availability of these data, which were used under license for this study, and so are not publicly available. Data are however available from the authors upon reasonable request and with the permission of the data providers.
