# Supplementary material for: Educational inequalities in hypothermia mortality in the Baltic countries and Finland in 2000–15
Source: Eur J Public Health. 2023 Apr 24;33(4):555–60. doi: 10.1093/eurpub/ckad062 (PMC10393481; doi:10.1093/eurpub/ckad062)
Supplement: ckad062_Supplementary_Data [file ckad062_supplementary_data.docx]

**Supplementary information**

**Supplementary Table 1** Impact of excluding register-only-based census records on hypothermia mortality among 30–74 year olds in Latvia, 2000–2015

| Sex | Period | Census + registry | Census |  | Census + registry | Census |  |
| --- | --- | --- | --- | --- | --- | --- | --- |
|  |  | ASMR (95% CI) | ASMR (95% CI) | *P* value | RR (95% CI) | RR (95% CI) | *P* value |
|  |  |  |  |  |  |  |  |
| Men | 2000–2007 | 25.0 (23.5–26.5) | 20.6 (19.2–22.1) | <0.000 | 1 | 1 |  |
|  | 2008–2015 | 17.1 (15.9–18.4) | 15.6 (14.4–16.9) | <0.000 | 0.68 (0.62–0.75) | 0.76 (0.68–0.84) | 0.125 |
|  |  |  |  |  |  |  |  |
| Women | 2000–2007 | 6.9 (6.3–7.7) | 5.8 (5.2–6.5) | <0.000 | 1 | 1 |  |
|  | 2008–2015 | 4.0 (3.4–4.5) | 3.5 (3.0–4.0) | 0.190 | 0.57 (0.48–0.67) | 0.59 (0.49–0.72) | 0.791 |

ASMR, age-standardised mortality rate per 100 000 person years; CI, confidence interval.

RR, rate ratios comparing the 2008–2015 period with the 2000–2007 period (reference category).

*P* values are for the differences resulting from excluding register-only-based census records.
